# Ultrasound-Enhanced Protective Effect of Tetramethylpyrazine against Cerebral Ischemia/Reperfusion Injury

**DOI:** 10.1371/journal.pone.0113673

**Published:** 2014-11-19

**Authors:** Chunbing Zhang, Fengmeng Teng, Juan Tu, Dong Zhang

**Affiliations:** 1 Department of Laboratory Medicine, Jiangsu Province of TCM, Nanjing, Jiangsu, P. R. China; 2 Basic Medical Sciences, Nanjing University of Chinese Medicine, Nanjing, Jiangsu, P. R. China; 3 Key Laboratory of Modern Acoustics (MOE), Institute of Acoustics, Nanjing University, Nanjing, Jiangsu, P. R. China; University of Naples Federico II, Italy

## Abstract

In traditional Chinese medicine, *Ligusticum wallichii* (Chuan Xiong) and its bioactive ingredient, tetramethylpyrazine (TMP), have been used to treat cardiovascular diseases and to relieve various neurological symptoms, such as those associated with ischemic injury. In the present study, we investigated whether ultrasound (US) exposure could enhance the protective effect of TMP against cerebral ischemia/reperfusion (I/R) injury. Glutamate-induced toxicity to pheochromocytoma (PC12) cells was used to model I/R injury. TMP was paired with US to examine whether this combination could alleviate glutamate-induced cytotoxicity. The administration of TMP effectively protected cells against glutamate-induced apoptosis, which could be further enhanced by US-mediated sonoporation. The anti-apoptotic effect of TMP was associated with the inhibition of oxidative stress and a change in the levels of apoptosis-related proteins, Bcl-2 and Bax. Furthermore, TMP reduced the expression of proinflammatory cytokines such as TNF-α and IL-8, which likely also contributes to its cytoprotective effects. Taken together, our findings suggest that ultrasound-enhanced TMP treatment might be a promising therapeutic strategy for ischemic stroke. Further study is required to optimize ultrasound treatment parameters.

## Introduction

Ischemic stroke, which results from the interruption of cerebral blood circulation, is characterized by the sudden development of neurological deficits in the damaged brain area [1]–[3]. Ischemia/reperfusion (I/R) injury, which is characterized by extensive neuronal apoptosis induced by inadequate oxygen supply, can happen to patients suffering ischemic diseases during/after the reperfusion treatment [4], [5]. It has been reported that the onset of post-ischemic apoptosis can be induced by multiple mechanisms. For example, oxidative stress caused by excessive levels of oxygen free radicals appears to play a key role in neuronal apoptosis [6]–[9]. Furthermore, post-ischemic inflammation is reported to be a major contributor to the delayed progression of cell death [10], [11]. The activation of inflammatory cells results in the production of pro-inflammatory and cytotoxic factors, including nitric oxide (NO), tumor necrosis factor-α (TNF-α), interleukin 1 (IL-1), interleukin 6 (IL-6) and reactive oxygen species (ROS) [11]–[16].

In traditional Chinese medicine, the herb *Ligusticum wallichii* (Chuan Xiong in Chinese) is commonly used to treat neurovascular and cardiovascular diseases. The compound 2,3,5,6-tetramethylpyrazine (TMP; C_8_H_12_N_2_; molecular weight, 136.2) is one of the most important active ingredients isolated from Chuan Xiong [17]. Over the past two decades, TMP has been demonstrated, in both animal experiments and clinical practice, to alleviate ischemic brain injury. TMP possesses diverse pharmacological properties, including the ability to dilate blood vessels, reduce arterial resistance and capillary permeability, increase cerebral blood flow, inhibit platelet aggregation and thrombosis, and improve microcirculation [18]–[21]. Accumulating evidence also shows that TMP helps maintain normal neuronal functions by preventing hypoxic and excitotoxic cell damage in hippocampal neurons [22] by scavenging free radicals, downregulating the production of nitric oxide [23], [24], and stimulating neuroprotective and anti-inflammatory processes after transient focal cerebral ischemia [25]. However, TMP displays low bioavailability *in vivo* and is metabolized rapidly, with a short half-life. It has been reported that even though TMP can traverse the blood-brain barrier quickly (within 10 min), it is rapidly eliminated from brain tissue. Furthermore, plasma levels of TMP are undetectable 2 h after intravenous administration in rats [26]–[28]. Therefore, optimizing the delivery and bioavailability of TMP to enhance its therapeutic efficacy is critical for the application of the drug in the clinic. The conventional solution is to raise the blood concentration of TMP to a relatively high level. However, this approach can produce undesired side-effects in patients (e.g. allergic reactions, angioedema, laryngeal edema, bronchial asthma or anaphylactic shock).

It has been demonstrated that with ultrasound (US) exposure, tiny pores can be transiently opened in cell membranes, through which macromolecules can be delivered into cells more easily. This process is called sonoporation [29]–[33]. There are usually small dissolved air bubbles (called cavitation nuclei) in liquid and tissues. These small bubbles, excited by ultrasound pulses with relatively higher pressure, may grow, oscillate and then collapse violently to result in inertial cavitation activity, which generates short-lived, non-specific pores in cell membranes [34]. Marmottant and Hilgenfieldt demonstrated dynamic vesicle deformation and lysis resulting from microstreaming and strain induced by low-amplitude US-driven bubble oscillations [35]. Thus, US-induced sonoporation is regarded as a promising non-invasive technique for enhancing gene and drug delivery efficiency, owing to its site specificity and the easy manipulation of US application parameters.

In this study, we investigated the feasibility of enhancing the protective effect of TMP for cerebral I/R injury using ultrasound exposure. We examined the cytoprotective effects of TMP combined with 1-MHz US exposure against glutamate-induced apoptosis in pheochromocytoma (PC12) cells. Immunohistochemistry assay, enzymatic rate assay, enzyme-linked immunosorbent assay (ELISA) and real-time polymerase chain reaction (PCR) were performed to examine changes in superoxide dismutase (SOD), lactate dehydrogenase (LDH), inflammatory factors (TNF-α, IL-8 and IL-6) and apoptosis, in an effort to provide insight into the mechanisms underlying the neuroprotective effects of TMP.

## Materials and Methods

### Chemicals and Materials

Dulbecco's modified eagle medium (DMEM) and fetal bovine serum (FBS) were purchased from Gibco (Grand Island, NY, USA). Glutamate and TMP were purchased from Sigma (St Louis, MO, USA) and were dissolved in dimethylsulfoxide (DMSO; Sigma) for all experiments. Cytokine (TNF-α, IL-6 and IL-8) ELISA kits were purchased from R&D (Minneapolis, MN, USA). Total RNA extraction kits and Trizol were purchased from Invitrogen (Carlsbad, CA, USA). Reverse transcription kits and PCR kits were purchased from Fermentas (Pittsburgh PA, USA). LDH and SOD assay kits were purchased from Nanjing Jian cheng (Jian cheng Bioengineering Institute, Nanjing, China). Cell apoptosis kit was purchased from BD Biosciences (San Jose, CA, USA). Penicillin-streptomycin was purchased from Wisent (Montreal, Canada). All other reagents were of analytical grade.

### Cell culture and treatment

PC12 cells were purchased from American Type Culture Collection (ATCC, USA). The cells were maintained in DMEM supplemented with 10% FBS and 1% penicillin-streptomycin solution at 37°C in a 5% CO_2_ incubator. To experimentally model apoptosis induced by cerebral I/R injury, cells were incubated with 12.5 mM glutamate for 12 h. To study the protective effect of TMP on PC12 cells, cells were pre-incubated with TMP for 3 h, and glutamate (12.5 mM) was then added for an additional 12 h of incubation. TMP and glutamate were dissolved in 50% DMSO; the concentration of DMSO in the final culture medium was <0.1%, which had no effect on cell viability. To study the impact of US on the cytoprotective effect of TMP, PC12 cells were treated with TMP (1 mM) and exposed to 1-MHz US at varying acoustic driving pressures for 20 s.

### Experimental groups

The experimental groups were categorized as follows: (1) Negative control group: PC12 cells were maintained in DMEM supplemented with 10% FBS and 1% penicillin-streptomycin solution; (2) Glutamate injury group (positive control): PC12 cells were treated for 12 h with DMEM containing 12.5 mM glutamate; (3) TMP group: PC12 cells were pretreated with medium containing 1 µM TMP for 3 h, and then incubated in medium containing 12.5 mM glutamate for 12 h; (4) US combined with TMP group: PC12 cells were cultured in DMEM containing TMP and exposed to US at varying acoustic peak negative pressures (P- = 0.05, 0.2, 0.5 or 1.0 MPa) for 20 s. After a 3-h treatment period, the cells were incubated in medium containing 12.5 mM glutamate for another 12 h.

### Ultrasound exposure system

**Figure 1:**
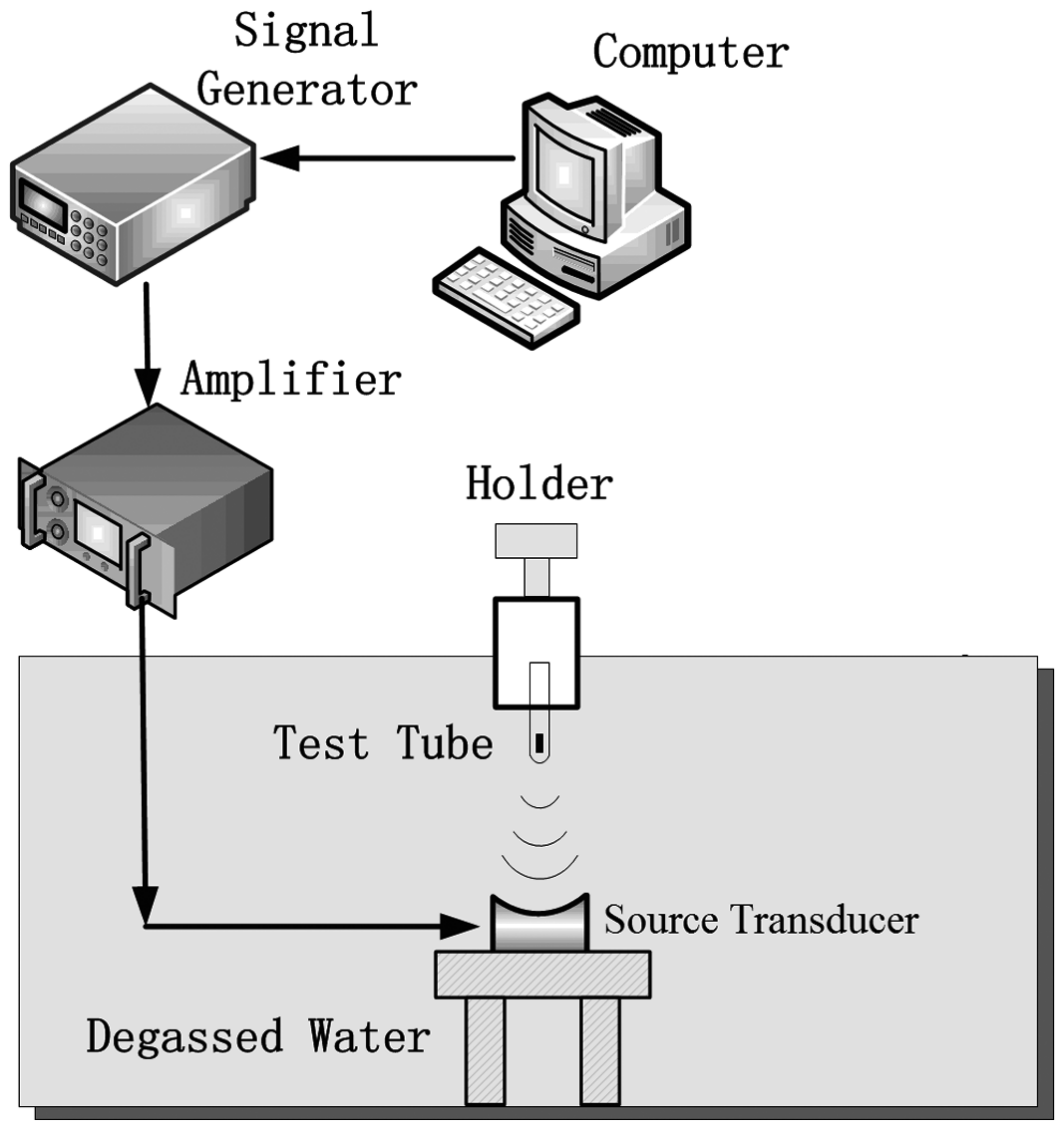
The schematic diagram of US-exposure system.

The US exposure apparatus is shown in Fig. 1. All experiments were performed in an acrylic tank filled with degassed water. An arbitrary waveform generator (Agilent 33250A, Santa Clara, CA, USA) supplied 1-MHz sinusoidal pulses at a fixed 20-cycle pulse length and 250-Hz pulse repetition frequency (PRF), with varied driving amplitudes. The output signals from the waveform generator were amplified through a RF power amplifier (ENI A150, Rochester, NY, USA) with a fixed gain of 50 dB, which were used to drive a 1-MHz self-made focused source transducer with a radius of 9.2 cm. The approximately 6.6-cm focal distance was about 6.6 cm and the −6 dB, focal width was ∼8 mm. A plastic test tube of 10-mm diameter and 50-mm length, filled with sample suspension (the liquid depth of the suspension was ∼16 mm), was capped with a custom-built rubber stopper that was used as an acoustic absorber to minimize the effect of standing waves, and then sealed with parafilm to minimize bacterial contamination. The test tube was aligned axially with the source transducer so that the center of the suspension was situated at the focal region. LabView (National Instruments, Austin, TX, USA) was used to control the waveform generator and the oscilloscope. The attenuation of the test tube wall was determined by measuring the US amplitude with/without placing the test tube *in situ*. Then, the *in situ* peak negative pressure of the source transducer was calibrated using a NTR needle hydrophone (TNU001A, NTR Systems, Inc., Seattle, WA, USA) with a 30-dB preamplifier (HPA30, NTR Systems, Inc., Seattle, WA, USA).

### Apoptosis rate assessed by flow cytometry

Twelve hours after glutamate injury, adherent PC12 cells were harvested and washed twice with pre-cooled PBS buffer. Then, the cells were incubated with 200 µl binding buffer containing 20 µl annexin V-FITC (20 µg/ml) and 10 µl propidium iodide (50 µg/ml) for 15 min at room temperature in the dark. Samples were detected in a FACSCanto II cell sorter (BD Biosciences, USA), and the data were analyzed with CellQuest software (BD Biosciences) [36].

### ELISA-based cytokine assays

Twelve hours after glutamate injury, cell supernatants were collected. After centrifugation at 3 000 r/min for 5 min, the cell supernatant was collected and stored at −80°C until analysis. The cell supernatant levels of proinflammatory cytokines (TNF-α, IL-6 and IL-8) were measured, respectively, using rat TNF-α, IL-6 and IL-8 ELISA kits (R&D Systems), according to the manufacturer's instructions.

### Immunoturbidimetric assay and enzymatic rate assay

Twelve hours after glutamate injury, cell supernatants were collected. After centrifugation at 3 000 r/min for 5 min, the cell supernatants were collected and stored at −80°C until analysis. The cell supernatant levels of two oxidative stress-related factors, SOD and LDH, were evaluated with immunoturbidimetric and enzymatic rate assay, respectively, according to the manufacturer's protocol.

### RNA isolation and real-time PCR analysis

Total RNA was extracted from PC12 cells using Trizol reagent (Invitrogen, USA) and reverse-transcribed to obtain single strand cDNA using the Reverse Transcription System (Fermentas, USA) according to the manufacturer's protocol. cDNA was amplified in a 20-µl reaction system containing 2 µl cDNA template, 1 µl probe (Invitrogen, Shanghai, China), 12.5 µl mix, 0.5 µM each of upstream and downstream primer, and double-distilled H_2_O. Real-time PCR was performed using the GeneAmp PCR system 9500 (Applied Biosystems, Foster City, CA). The following primer sequences (Invitrogen, Shanghai, China) were used: Bcl-2 (sense: 5′-TGCGCTCAGCCCTGTG-3′, antisense: 5′-GGTAGCGACGAGAGAAGTCATC-3′, probe: 5′-CCACCTGTGGTCCACCTG-3′); Bax (sense: 5′-CAAGAAGCTGAGCGAGTGTCT-3′, antisense: 5′-CAATCATCCTCTGCAGCTCCATATT-3′, probe: 5′-CCAGTTCATCTCCAATTCG-3′); GAPDH (sense: 5′-GTGCCAAAAGGGTCATCATCTC-3′, antisense: 5′-GGTTCACACCCATCACAAACATG-3′, probe: 5′-TTCCGCTGATGCCCC-3′). The thermocycling parameters were as follows: 3 min at 95°C; 40 cycles of 95°C for 20 s, 52°C for 35 s, and 72°C for 10 s. The 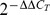 analysis method was used to quantify relative amounts of mRNA. For normalization of gene expression levels, the result was expressed as fold change relative to control. mRNA ratios relative to the housekeeping gene GAPDH were calculated.

### Morphological analysis using scanning electron microscopy

To examine the effects of US exposure on cell membranes, cells were imaged with scanning electron microscopy (SEM) at 10 000× magnification. It has been reported that US-induced cell membrane damage is rapidly repaired. Zarnitsyn et al. theoretically calculated that the membrane cavity (∼300 nm radius) is resealed with a half time of 20–50 s [37]. Thus, after US exposure, the cells were immediately (∼5–10 s) fixed with 2.5% glutaraldehyde solution for 2 h at 4°C, and then washed twice in PBS. Cells were then subjected to graded alcohol dehydration in 33, 50, 60, 80, 90 and 100% ethanol for 20 min each. Each step was repeated twice. After lyophilization (Freezone 6 Freeze Dry System, Labconco Co., Kansas City, Missouri, USA), each sample was gold sputter-coated for 5 min at 125 mA in an argon atmosphere with an approximately 50-nm-thick coating (Emitech K550X. Sputter Coating Systems, England). A field emission scanning electron microscope (JSM-5610LV, JEOL Ltd., Tokyo, Japan) was used with a gun acceleration voltage of 15 kV and a working distance of 8 mm.

### Statistical analysis

All data were expressed as the mean ± standard deviation (SD) of 5 replicate treatments. In order to detect significant differences among experimental groups, analysis of variance (ANOVA) was performed based on Newman-Keuls method. A *p*<0.05 was considered statistically significant. The statistic analyses were performed using SPSS software (IBM Corporation, Armonk, NY, USA).

## Results and Discussion

### US-enhanced protective effect of TMP on glutamate-induced apoptosis

**Figure 2:**
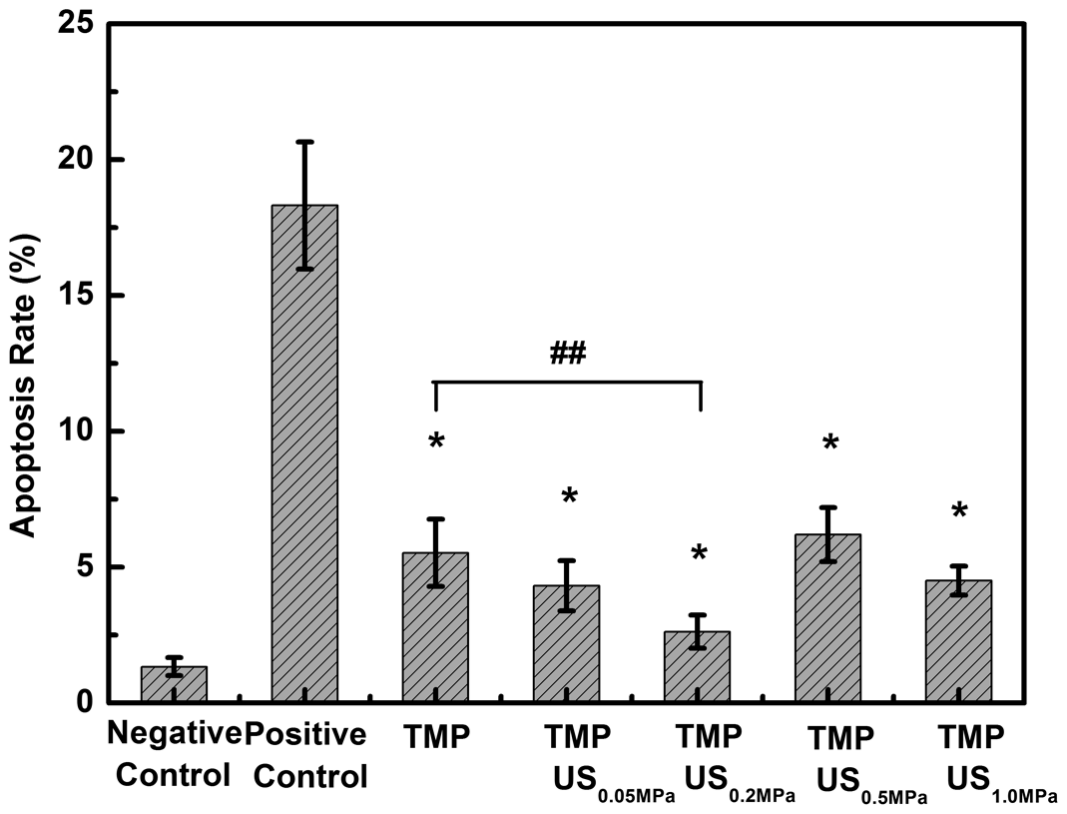
Effect of TMP combined with US exposure on the apoptosis rate of glutamate-damaged PC12 cells. Each bar represents the mean plus or minus the standard deviation (n = 5, *p<0.01 compared with the positive control group; ^##^ *p*<0.05 compared with the TMP group).

Previous studies demonstrate that TMP can exert a significant protective effect against cerebral ischemic injury, as shown by its ability to reduce cerebral apoptosis [22]–[25], [38]. To quantitatively evaluate the protective effect of TMP combined with US exposure on PC12 cells, the apoptosis rate was assessed by FITC-labeled annexin V/PI double staining and flow cytometry analysis. As shown in Fig. 2, compared with the negative control group, which had an apoptosis rate of 1.34±0.33%, a substantial increase in the apoptosis rate was observed in PC12 cells treated with 12.5 mM glutamate for 12 h (18.32±2.34%). If these glutamate-treated cells were pretreated with 1 mM TMP for 3 h, the apoptosis rate decreased to 5.6±1.2%, which suggests that TMP can effectively protect cells against glutamate-induced cytotoxicity. To examine whether the protective effect of TMP can be further enhanced with combined US exposure, the suspension of glutamate-treated cells and TMP was sonicated with 1-MHz US for 20 s, with varying acoustic driving pressures. The apoptosis rate was substantially lowered to 2.6±0.6% when the acoustic pressure was increased to 0.2 MPa. When a relatively high acoustic pressure was applied (0.5 and 1.0 MPa), the apoptosis rate reverted to a level comparable to that in the group treated with TMP only (6.20±0.99% and 4.50±0.54% for 0.5 and 1.0 MPa, respectively) as a result of the cell damage caused by excessive acoustic energy. The results clearly show that US exposure of an appropriate acoustic intensity can substantially enhance the neuroprotective effect of TMP against glutamate-induced cytotoxicity in PC12 cells.

### Combined effect of TMP and US on oxidative stress-related enzymes

There is increasing evidence that oxidative stress, an imbalance between the production of ROS and the biological system's ability to detoxify them, may be a key factor in the pathophysiology of cardiovascular and cerebrovascular diseases [6]–[9]. In the present study, glutamate was used to induce oxidative stress, which can damage DNA and trigger the peroxidation of lipids and proteins in the cell membrane. Thus, the combined protective effect of TMP and US might result from the inhibition of glutamate-induced oxidative stress. Here, the expression levels of oxidative stress-related factors, LDH, SOD and Bcl-2/Bax, were assessed by enzymatic rate assay, immunoturbidimetric assay and real-time PCR, respectively.

**Figure 3:**
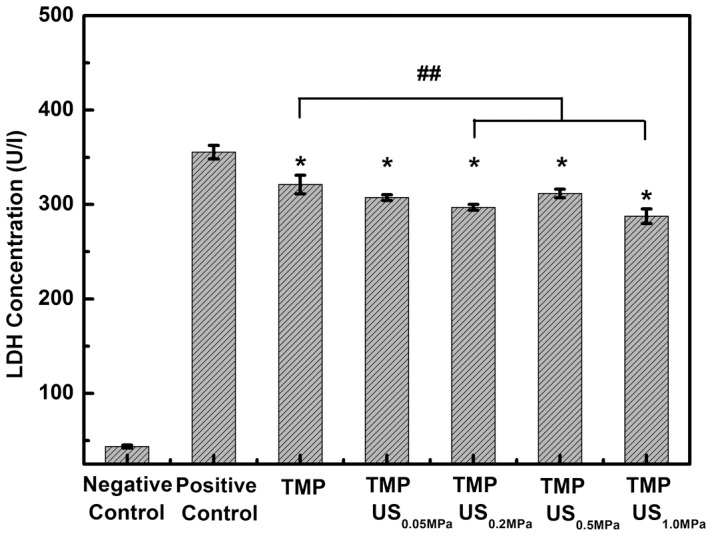
Effect of TMP combined with US exposure on glutamate-induced release of LDH in PC12 cells. Each bar represents the mean plus or minus the standard deviation (n = 5, *p<0.01 compared with the positive control group; ^##^ *p*<0.05 compared with the TMP group).

LDH is of significance because it is an enzyme released during tissue damage. An increase in LDH is a typical indicator of common injuries and diseases [7]. As shown in Fig. 3, a significant increase in LDH release was observed for glutamate-damaged cells (355.4±7.0 U/l). With TMP treatment, less LDH was detected (321.1±9.7 U/l), suggesting that treatment with TMP can alleviate cell damage caused by glutamate. When combined with increasing acoustic driving pressure, from 0.05 to 1.0 MPa, LDH release decreased from 317.3±3.5 U/l to 287.6±7.7 U/l, which suggests that the protective effect of TMP can be improved by US exposure.

**Figure 4:**
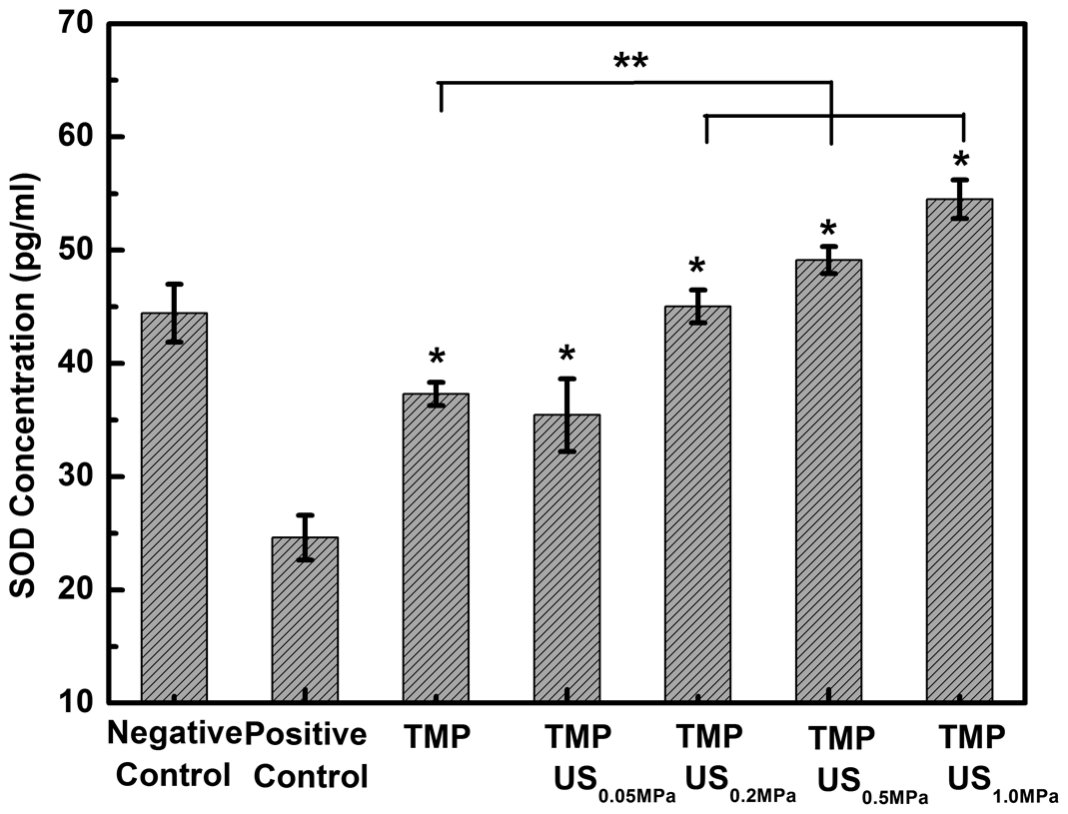
Effect of TMP combined with US exposure on glutamate-induced release of SOD in PC12 cells. Each bar represents the mean plus or minus the standard deviation (n = 5, *p<0.01 compared with the positive control group; **p<0.01 compared with the TMP group).

Another antioxidant enzyme, SOD, has been reported previously to be involved in antioxidant defense in vascular endothelial cells [7], [9], [39]. SOD can scavenge intracellular ROS by converting them to hydrogen peroxide, which can be further converted to water. However, the activity of SOD can be impaired in patients with ischemia/reperfusion brain injury [40]. As shown in Fig. 4, the SOD level in the negative control group was 44.4±2.5 pg/ml. By adding glutamate, the SOD level was suppressed to 24.6±1.9 pg/ml. The decline in SOD content could be prevented by the use of TMP (37.3±1.0 pg/ml). Moreover, the combination of TMP and US exposure elevated SOD content from 35.4±3.2 to 54.5±1.7 pg/ml, as P- increased from 0.05 to 1.0 MPa. This observation suggests that the cytoprotective effect of TMP combined with US on glutamate-induced oxidative stress might be related to the reduction of intracellular ROS via the enhancement of endogenous antioxidative mechanisms (i.e. increased SOD expression).

### Combined effect of TMP and US on mitochondrial apoptosis regulatory genes

**Figure 5:**
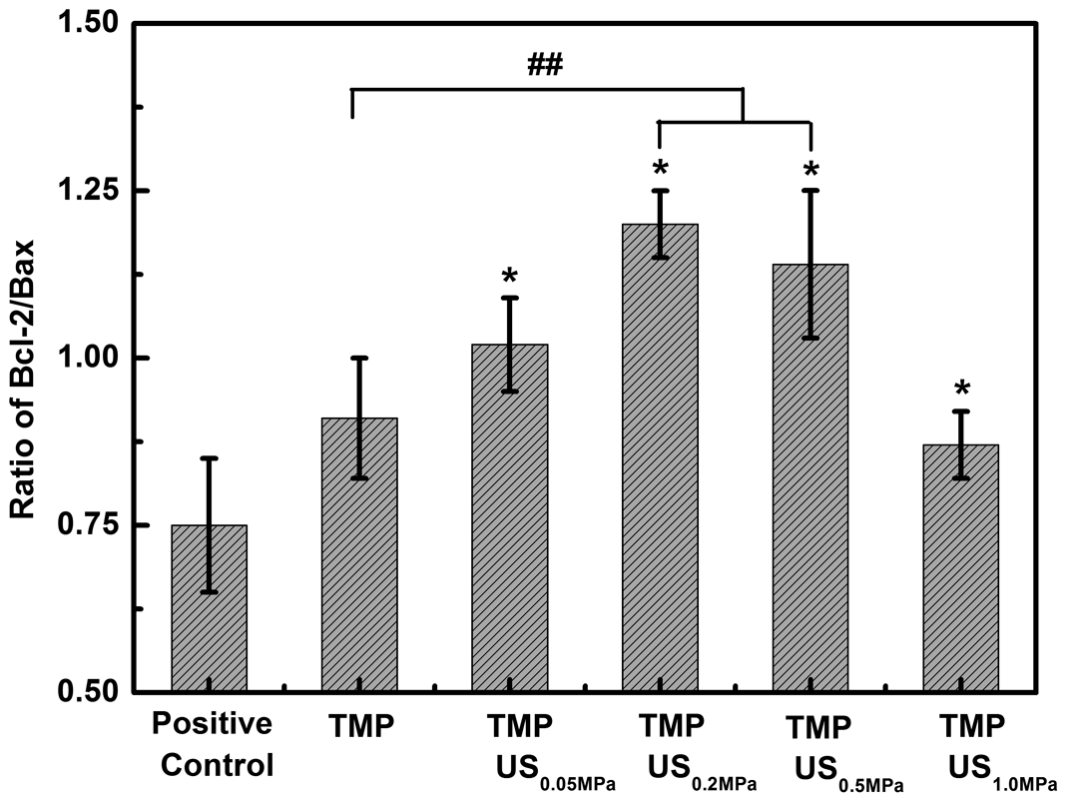
Effect of TMP combined with US exposure on the ratio of Bcl-2/bax measured for glutamate-damaged PC12 cells. Each bar represents the mean plus or minus the standard deviation (n = 5, *p<0.01 compared with the positive control group; ^##^ *p*<0.05 compared with the TMP group).

The overproduction of ROS can also break down mitochondrial function via formation of the mitochondrial permeability transition pore, which is an early event in apoptosis [41]. The apoptotic process involves numerous genes and proteins. The Bcl-2 family is a major apoptotic regulator [38], [42]. The Bcl-2 family of proteins can act as sensors to integrate death and survival signals in the mitochondrial pathway. Bcl-2 serves as a potent cell death suppressor, and apoptosis can be prevented by overexpression. In contrast, Bax is a key factor that promotes cell death. The translocation of Bax to the mitochondrial membrane can result in the loss of mitochondrial membrane potential and an increase in mitochondrial permeability, which in turn triggers apoptosis. In the present study, the impact of TMP treatment combined with US on the cellular Bcl-2/Bax ratio was evaluated using quantitative RT-PCR analysis. Figure 5 shows the results obtained using glutamate-damaged PC12 cells treated with TMP combined with US exposure at different acoustic pressures. The increase in the ratio of Bcl-2/Bax (from 0.91±0.09 to 1.20±0.15), which indicates an up-regulation of Bcl-2 and a down-regulation of Bax, is clearly observed when P- is raised from 0 to 0.2 MPa. This observation suggests that TMP can provide neuroprotection to PC12 cells against oxidative stress induced brain injury by blocking the mitochondrial apoptotic pathway, which agrees well with previous reports [38]. Consistent with the results obtained for the cell apoptosis rate, the Bcl-2/Bax ratio drops from 1.20±0.15 to 0.87±0.05 as P- increases from 0.2 to 1.0 MPa, suggesting that excessive applied acoustic energy might hinder the neuroprotective effect of TMP treatment.

### Combined effect of TMP and US on inflammatory factors

In addition to its anti-apoptotic properties, TMP might also protect neuron cells against ischemic injury by inhibiting the inflammatory reaction. Cytokines are a diverse category of small glycoproteins (∼5–25 kDa) released by different types of cells, such as endothelial cells, immune cells and platelets, and function as intercellular messengers that regulate inflammatory and immunologic responses [9]. Previous studies have reported that proinflammatory cytokines (e.g. TNF-α, IL-6) might be elevated in serum in the early phase of acute ischemic stroke [9], [14], [15]. Thus, to examine whether TMP protects against cerebral ischemia/reperfusion injury by modulating the inflammatory response, the release of inflammation-related factors (TNF-a, IL-6, IL-8) were examined in PC12 cells using ELISA assays.

**Figure 6:**
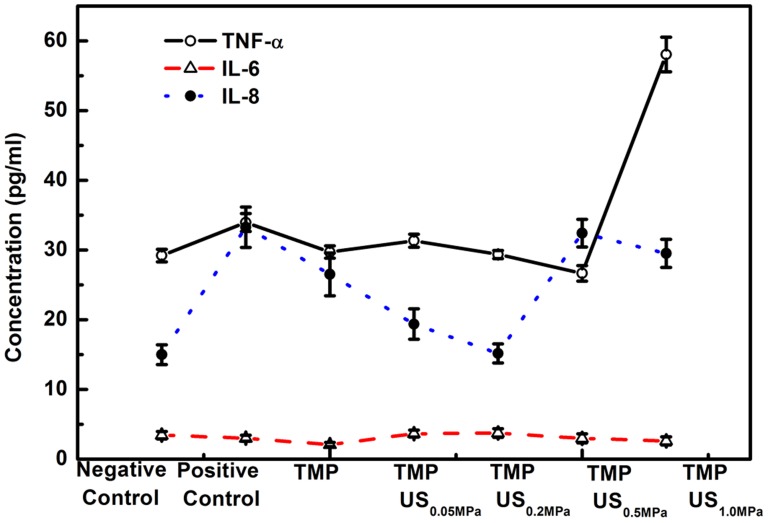
Effect of TMP combined with US exposure on the expression of inflammatory-related factors.

As shown in Fig. 6, after experimentally modeling cerebral ischemia/reperfusion injury by pre-treating PC12 cells with glutamate, a robust increase in IL-8 (from 15.0±1.4 to 33.3±2.9 pg/ml) can be observed. In the groups treated with TMP combined with US, the IL-8 concentration was significantly reduced to 15.1±1.4 pg/ml, as the acoustic driving pressure was raised to 0.2 MPa. With higher acoustic pressures (e.g. P->0.5 MPa), the IL-8 concentration increases again (to 32.4±2.0 pg/ml). A similar trend could be observed for TNF-α, although the change was smaller than that for IL-8. Previous studies have demonstrated that both IL-8 and TNF-α act as typical proinflammatory cytokines, and an elevation in TNF-α and IL-8 levels can be detected after cerebral ischemic injury [9], [15], [43]. Therefore, the current results indicate that the neuroprotective effect of TMP is also, in part, mediated by its anti-inflammatory properties, and that it can effectively down-regulate the expression of some proinflammatory cytokines (e.g. IL-8 and TNF-α) after ischemic brain injury. Moreover, similar to its anti-apoptotic properties, the anti-inflammatory properties of TMP can also be enhanced by US exposure with an appropriate driving pressure.

In contrast to IL-8 and TNF-α, no obvious change is observed for IL-6 (as shown Fig. 6). Lindsberg et al [12] reported that IL-6 shares some of the biological activities of IL-1 in the development of inflammatory responses, while it is also believed that IL-6 should have anti-inflammatory properties because of its ability to induce IL-1ra synthesis [13]. Thus, it is unclear whether IL-6 is more pro- or anti-inflammatory. The current results suggest that the neuroprotective effects of TMP are not associated with changes in IL-6 levels.

### US-induced sonoporation on cell membranes

**Figure 7:**
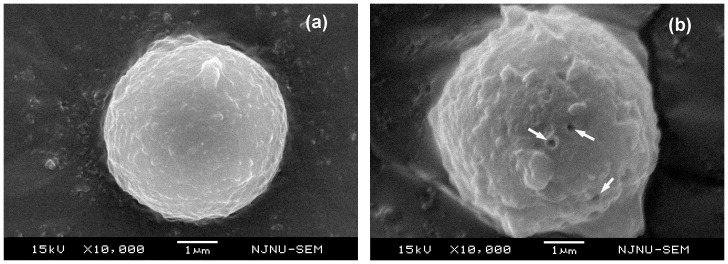
Morphology of sample cell (a) without US exposure and (b) exposed to US working at 1-MH central frequency, 20-s exposure duration, 20-cycle pulse length, and 0.5-MPa acoustic driving pressure. The white arrows point out some sonoporation pores.

The preceding results demonstrate that the neuroprotective effect of TMP can be effectively enhanced by combining with US exposure (e.g. P- = 0.2 MPa), although ultrasonic sonication of relatively high intensity might attenuate TMP protection. US-induced sonoporation can transiently enhance cell membrane permeability to facilitate the uptake of foreign genes and drugs into cells [29]–[35]. Therefore, electron microscopy studies were applied to examine the membrane morphology of PC12 cells treated with/without US exposure. As shown in Fig. 7a, without US exposure, the cell has a spherical shape with a relatively intact surface. However, after US exposure, tiny pores and a rougher membrane surface can be observed (Fig. 7b). Qiu et al reported that the diameter of the sonoporation pore is highly correlated with US-induced cavitation intensity, which is dependent on the acoustic driving pressure [44]. For the acoustic intensity within a certain range, the increase in P- can benefit gene/drug delivery to cells by improving the permeability of cell membrane to allow more TMP entering the cells. However, if the US driving pressure exceeds a certain level (P->0.5 MPa), sonoporation pores with an excessively large diameter could be generated on the cell membrane, which might induce permanent damage, resulting in cell apoptosis. Therefore, appropriate US driving parameters should be employed to optimize the intracellular uptake of TMP.

## Conclusion

In this study, we show that the combination of TMP and US can effectively protect PC12 cells from glutamate-induced oxidative damage, which models neuronal injury following I/R. The neuroprotective effects of TMP appear to be mediated by its ability to inhibit cell apoptosis by reducing cellular oxidative stress and impacting levels of critical apoptosis-related gene factors. TMP also has anti-inflammatory properties and inhibits the expression of proinflammatory cytokines such as TNF-α and IL-8, which likely also contributes to the cytoprotective effects. Collectively, our results suggest that with optimal acoustic driving parameters, US-enhanced TMP treatment might have therapeutic efficacy in patients with I/R injury.

## References

[1] Virmani R, Ladich ER, Burke AP, Kolodqie FD (2006) Histopathology of carotid atherosclerotic disease. Neurosurgery 59: S219–227. 17053606 10.1227/01.NEU.0000239895.00373.E4

[2] Behrouz R, Malek AR, Torbey MT (2012) Small Vessel Cerebrovascular Disease: The past, present, and Future. Stroke Res Treat 2012: 839151–839158. 22315706 10.1155/2012/839151 PMC3270464

[3] Sahota P, Savitz SI (2011) Investigational therapies for ischemic stroke: neuroprotection and neurorecovery. Neurotherapeutics 8: 434–451. 21604061 10.1007/s13311-011-0040-6 PMC3250280

[4] Eltzschig HK, Eckle T (2011) Ischemia and reperfusion–from mechanism to translation. Nat Med 17: 1391–1401. 22064429 10.1038/nm.2507 PMC3886192

[5] Genovese T, Mazzon E, Paterniti I, Esposito E, Bramanti P, et al (2011) Modulation of NAPDH oxides activation in cerebral ischemia/reperfusion injury in rats. Brain Res 1372: 92–102 (2011). 21138737 10.1016/j.brainres.2010.11.088

[6] Heo JM, Kim HJ, Ha YM, Park MK, Kang YJ, et al (2007) YS 51, 1-(beta-naphtylmethy)-6,7-dihydroxy-1,2,3,4-tetrahydroisoquinoline, protects endothelial cells against hydrogen peroxide-induced injury via barbon monoxide derived from heme oxygenase-1. Biochem Pharmacol 74: 1361–1370. 17719563 10.1016/j.bcp.2007.07.023

[7] Ou Y, Guo XL, Zhai L, Liu XY, Cheng YN (2010) Tyrazine derivative, protects vascular endothelial cells from oxidation damage by hydrogen peroxide,. Pharmazie 65: 755–759. 21105578

[8] Ito Y, Ohkubo T, Asano Y, Hattori K, Shimazu T, et al (2010) Nitric oxide production during cerebral ischemia and reperfusion in eNOS-and nNOS-knockout mice. Curr Neurovasc Res 7: 23–31. 20158465 10.2174/156720210790820190

[9] Zhang H, Sun R, Liu XY, Shi XM, Wang WF, et al (2014) A tetramethylppiperazine derivate CXC1 prevents cell injury in SH-SY5Y cells and improves memory dfunction of rates with vascular dementia,. Neurochem Res 39: 276–286. 24357351 10.1007/s11064-013-1219-5

[10] Iadecola C, Anrather J (2011) The immunology of stroke: from mechanisms to translation. Nat Med 17: 796–808. 21738161 10.1038/nm.2399 PMC3137275

[11] Huang J, Upadhyay UM, Tamargo RJ (2006) Inflammation in stroke and focal cerebral ischemia. Surg Nerology 66: 232–245. 10.1016/j.surneu.2005.12.028 16935624

[12] Lindsberg PJ, Carpen O, Paetau A, Karjalainen-Lindsberg ML, Kaste M (1996) Endothelial ICAM-1 expression associated with inflammatory cell respons in human ischemic stroke. Circulation 94: 939–945. 8790029 10.1161/01.cir.94.5.939

[13] Relton JK, Martin D, Thompson RC, Russell DA (1996) Peripheral administration of INterleukin-1 receptor antagonist inhibits brain damage after focal cerebral ischemia in the rat. Exp Neurol 138: 206–213. 8620919 10.1006/exnr.1996.0059

[14] Oto J, Suzue A, Inui D, Fukuta Y, Hosotsubo K, et al (2008) Plasma proinflammatory and anti-inflammatory cytokine and catecholamine concentrations as predictors of neurological outcomes in acute stoke patients. J Anesthes 22: 207–212. 10.1007/s00540-008-0639-x 18685925

[15] Tu X, Kang WZ, Shi SS, Chen Y, Wang CH, et al (2011) Baicalin inhibits TLR2/4 Signaling pathway in rat brain following permanent cerebral ischemia. Inflammation 34: 463–470. 20859668 10.1007/s10753-010-9254-8

[16] Lambertsen KL, Biber K, Finsen B (2012) Inflammatory cytokines experimental and human stroke. J Cereb Blood Flow Metab 32: 1677–1698 (2012). 22739623 10.1038/jcbfm.2012.88 PMC3434626

[17] Lui C (1978) Simplified review of the effects of Chinese medical herbals on the cardiovascular system. Clin Med Res 4: 24–37.

[18] Ho WK, Wen HL, Lee CM (1989) Tetramethylpyrazine for treatment of experimentally induced stroke in Mongolian gerbils. Stroke 20: 96–99. 2911841 10.1161/01.str.20.1.96

[19] Liao SL, Raung SL, Chen CJ (2002) Japanese encephalitis virus stimulates superoxide dismutase activity in rat glial cultures. Neurosci Lett 324: 133–136. 11988345 10.1016/s0304-3940(02)00236-7

[20] Dai XZ, Bache RJ (1985) Coronary and systemic haemodynamic effect of tetramethylpyrazine in the dog. J Cardiovasc Pharmacol 7: 841–849. 2413290 10.1097/00005344-198509000-00005

[21] Tuttle RS, Marmelstein L, Trad T, Reddy S, Radley T (1989) In vitro uterine response to tetramethylpyrazine, the active constituent of chung chong (a traditional Chinese medicine). Am J Obstet Gynecol 161: 1319–1323. 2589459 10.1016/0002-9378(89)90691-1

[22] Shih YH, Wu SL, Chiou WF, Ku HH, Ko TL, et al (2002) Protective effects of tetramethylpyrazine on kainite-induced excitotoxicity in hippocampal culture. Neuroreport 13: 515–519. 11930173 10.1097/00001756-200203250-00032

[23] Zhang Z, Wei T, Hou J, Li G, Yu S, et al (2003) Tetramethylpyrazine scavengers superoxide anion and decreases nitric oxide production in human polymorphnuclear leukocytes. Life Sci 72: 2465–2472. 12650854 10.1016/s0024-3205(03)00139-5

[24] Zhao H, Zhuang F, Stoltz JF, Wang X (2003) Comparative studies of LFA-1/ICAM-1 interaction by micropipette and flow chamber techniques. Biorheology 40: 179–187. 12454403

[25] Liao SL, Kaob TK, Chen WY, Lina YS, Chen SY, et al (2004) Tetramethylpyrazine reduces ischemic brain injury in rats. Neuroscience Letters 372: 40–45. 15531085 10.1016/j.neulet.2004.09.013

[26] Liang CC, Hong CY, Chen CF, Tsai TH (1999) Measurement and pharmacokinetic study of tetramethylpyrazine in rat blood and its regional brain tissue by highperformance liquid chromatography. J Chromatogr B Biomed Sci Appl 724: 303–309. 10219672 10.1016/s0378-4347(99)00010-9

[27] Tsai TH, Liang C (2001) Pharmacokinetics of tetramethylpyrazine in rat blood and brain using microdialysis. Int J Pharm 216: 61–66. 11274807 10.1016/s0378-5173(01)00572-5

[28] Mei D, Mao S, Sun W, Wang YJ, Kissel T (2008) Effect of chitosan structure properties and molecular weight on the intranasal absorption of tetramethylpyrazine phosphate in rats. Eur J Pharm Biopharm 70: 874–881. 18656537 10.1016/j.ejpb.2008.06.031

[29] Ferrara K, Pollard R, Borden M (2007) Ultrasound Microbubble Contrast Agents: Fundamentals and Application to Gene and Drug Delivery. Annu Rev Biomed Eng 9: 415–447. 17651012 10.1146/annurev.bioeng.8.061505.095852

[30] Hynynen K (2008) Ultrasound for drug and gene delivery to the brain. Advanced Drug Delivery Rev 60: 1209–1217. 10.1016/j.addr.2008.03.010 PMC2504014 18486271

[31] Mitragotri S (2005) Healing sound: the use of ultrasound in drug delivery and other therapeutic applications. Nat Rev Drug Discov 4: 255–260. 15738980 10.1038/nrd1662

[32] Newman C, Bettinger T (2007) Gene therapy progress and prospects: ultrasound for gene transfer. Gene Ther 14: 465–475. 17339881 10.1038/sj.gt.3302925

[33] Van Wamel A, Kooiman K, Harteveld M, Emmer M, ten Cate FJ, et al (2006) Vibrating microbubbles poling individual cells: drug transfer into cells via sonoporation. J. Control. Release 112: 149–155. 10.1016/j.jconrel.2006.02.007 16556469

[34] May DJ, Allen JS, Ferrara KW (2002) Dynamics of and fragmentation of thick-shelled microbubbles. IEEE Trans Ultra Ferro, Freq Control 49: 1400–1410. 10.1109/tuffc.2002.1041081 12403141

[35] Marmottant P, Hhilgenfeldt S (2003) Controlled vesicle deformation and lysis by single oscillating bubbles. Nature 423: 153–156. 12736680 10.1038/nature01613

[36] Ilan Z, Eldad M, Nurit N, Drorit L, Anat A, et al (1994) Dopamine induces apoptosis-like cell death in cultured chick sympathetic neurons d A possible novel pathogenetic mechanism in Parkinson's disease. Neurosci Lett 170: 136e40. 8041491 10.1016/0304-3940(94)90258-5

[37] Zamitsyn V, Rostad C, Prausnitz M (2008) Modeling transmembrane transport through cell membrane wounds created by acoustic cavitation. Biophys J 95: 4124–4138. 18676653 10.1529/biophysj.108.131664 PMC2567961

[38] Chen XR, Zhang L, Hu JJ, Sun L, Du GH (2007) Nerroprotective effects of tetramethylpyrazine on dydrogen peroxide-induced apoptosis in PC12 cells, Cell Biol. Internation 31: 438–443. 10.1016/j.cellbi.2006.10.001 17321170

[39] Muzykantov VR (2001) Targeting of superoxide dismutase and catalase to vascular endothelium. J. Control. Release 71: 1–21. 10.1016/s0168-3659(01)00215-2 11245904

[40] Nita DA, Nita V, Spulber S, Moldovan M, Popa DP, et al (2001) Oxidative damage following cerebral ischemia depends on reperfusion-a biochemical study in rat. J Cell Mol Med 5: 163–170. 12067499 10.1111/j.1582-4934.2001.tb00149.x PMC6738122

[41] Hausenloy DJ, Yellon DM (2003) The mitochondrial permeability transition pore: its fundamental role in mediating cell death during ischaemia and reperfusion. J Mol Cell Cardiol 35: 339–341. 12689812 10.1016/s0022-2828(03)00043-9

[42] Pias EK, Aw TY (2002) Early redox imbalance mediates hydroperoxide-induced apoptosis in mitotic competent undifferentiated PC12 celss. Cell Death Differ 9: 1007–1016. 12181751 10.1038/sj.cdd.4401064

[43] Kostulas N, Kivisakk P, Huang Y, Matusevicius D, Kostulas V, et al (1998) Ischemic stroke is associated with a systemic increase of blood monouclear cells expressing interleukin-8 mRNA,. Stroke 29: 462–466. 9472890 10.1161/01.str.29.2.462

[44] Qiu YY, Luo Y, Zhang YL, Cui WC, Zhang D, et al (2010) The correlation between acoustic cavitation and sonoporation involved in ultrasound-mediated DNA transfection with polyethylenimine (PEI) in vitro. J Control Release 145: 40–48. 20398711 10.1016/j.jconrel.2010.04.010

